# Glutamate Racemase Is the Primary Target of β-Chloro-d-Alanine in Mycobacterium tuberculosis

**DOI:** 10.1128/AAC.01249-16

**Published:** 2016-09-23

**Authors:** Gareth A. Prosser, Anne Rodenburg, Hania Khoury, Cesira de Chiara, Steve Howell, Ambrosius P. Snijders, Luiz Pedro S. de Carvalho

**Affiliations:** a Mycobacterial Metabolism and Antibiotic Research Laboratory, The Francis Crick Institute, London, United Kingdom; b Proteomics Scientific Technology Platform, Mill Hill Laboratory, The Francis Crick Institute, London, United Kingdom

## Abstract

The increasing global prevalence of drug resistance among many leading human pathogens necessitates both the development of antibiotics with novel mechanisms of action and a better understanding of the physiological activities of preexisting clinically effective drugs. Inhibition of peptidoglycan (PG) biosynthesis and cross-linking has traditionally enjoyed immense success as an antibiotic target in multiple bacterial pathogens, except in Mycobacterium tuberculosis, where it has so far been underexploited. d-Cycloserine, a clinically approved antituberculosis therapeutic, inhibits enzymes within the d-alanine subbranch of the PG-biosynthetic pathway and has been a focus in our laboratory for understanding peptidoglycan biosynthesis inhibition and for drug development in studies of M. tuberculosis. During our studies on alternative inhibitors of the d-alanine pathway, we discovered that the canonical alanine racemase (Alr) inhibitor β-chloro–d-alanine (BCDA) is a very poor inhibitor of recombinant M. tuberculosis Alr, despite having potent antituberculosis activity. Through a combination of enzymology, microbiology, metabolomics, and proteomics, we show here that BCDA does not inhibit the d-alanine pathway in intact cells, consistent with its poor *in vitro* activity, and that it is instead a mechanism-based inactivator of glutamate racemase (MurI), an upstream enzyme in the same early stage of PG biosynthesis. This is the first report to our knowledge of inhibition of MurI in M. tuberculosis and thus provides a valuable tool for studying this essential and enigmatic enzyme and a starting point for future MurI-targeted antibacterial development.

## INTRODUCTION

Mycobacterium tuberculosis, the causative agent of pulmonary tuberculosis, is a human pathogen of serious global significance, having been responsible for over 1.3 million deaths worldwide in 2012 alone (1). Despite an established curative treatment being available for drug-sensitive infections, the complexity (4-drug cocktail), length of treatment (6 months), and associated side effects of this therapy limit its ultimate effectiveness and are at least partially responsible for the increasing incidence of clinical drug resistance. These factors and others underscore the exceptional requirement for new antibiotics with novel mechanisms of action to treat this recalcitrant and persistent disease.

While highly exploited in other bacteria, inhibition of peptidoglycan (PG) biosynthesis has thus far achieved poor success as a therapeutic strategy in M. tuberculosis. The only compound clinically approved for treatment of tuberculosis to target this pathway is d-cycloserine (DCS), a structural analogue of d-alanine that inhibits d-alanine:d-alanine ligase (Ddl) and alanine racemase (Alr), enzymes involved in the cytoplasmic (soluble) stages of PG biosynthesis (2, 3). In light of the unique mechanism of action of this antibiotic, its potency against M. tuberculosis, and sparse reports of clinical resistance, we have been studying the molecular and cellular enzymology of the interactions of DCS with the M. tuberculosis target orthologues in order to potentially design improved drug candidates (4–6).

**FIG 1:**
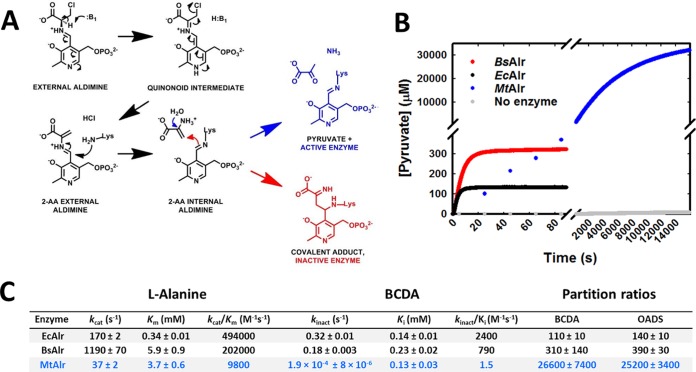
BCDA is a poor inactivator of MtAlr. (A) Currently accepted molecular model of BCDA hydrolysis or covalent adduct formation catalyzed by PLP-dependent enzymes. (B) Representative time course kinetics of pyruvate formation of recombinant *Ec*Alr, *Bs*Alr, and *Mt*Alr (normalized to 1 μM enzyme) in the presence of 1 mM BCDA. Reactions were monitored by observing NADH oxidation upon the production of pyruvate from BCDA via a coupled enzyme assay system (lactate dehydrogenase). (C) Steady-state kinetic and time-dependent inhibition parameters for Alr orthologues with l-alanine and BCDA. Results are the averages ± standard errors of the means (SEM) from at least triplicate data sets. See also Fig. S1 in the supplemental material.

To more thoroughly understand drug-target engagement within the d-alanine pathway, we have been studying the enzymology and microbiology of alternative Ddl and Alr inhibitors, including the Alr inhibitor β-chloro–d-alanine (BCDA). Previous studies employing a variety of Alr orthologues have demonstrated a unique mechanism of covalent inhibition for this compound (Fig. 1A; see also Fig. S1A in the supplemental material) (7–9). Following binding to the Alr active site and Schiff base formation with the enzyme's pyridoxal 5′-phosphate (PLP) prosthetic group, BCDA undergoes base-catalyzed deprotonation at the alpha position followed by spontaneous elimination of the β-chloride substituent. The resulting 2-amino acrylate (2-AA) intermediate is then presumed to dissociate from the enzyme active site and, in the majority of turnovers, to hydrolyze nonenzymatically to its keto-acid product, pyruvate. However, due to the reactivity of 2-AA (an electrophile), the intermediate is attacked back in a proportion of turnovers and covalently modifies the PLP-lysine internal aldimine linkage within the Alr active site, leading to irreversible enzyme inactivation. Importantly, BCDA shows potent antibacterial (including antimycobacterial) activity (10, 11), revealing it to be an ideal candidate for studying drug-induced and selective inhibition of Alr within a whole-cell system. However, during our initial studies performed with BCDA (presented here), we noticed very poor inhibition activity against M. tuberculosis Alr (*Mt*Alr) *in vitro*, despite robust activity against other Alr orthologues. This report describes our further investigation of this phenomenon, which ultimately resulted in the discovery of BCDA as an irreversible inhibitor of M. tuberculosis glutamate racemase (MurI) both *in vitro* and at the whole-cell level. To the best of our knowledge, this is the first report of a MurI-targeting compound with whole-cell activity against M. tuberculosis that therefore represents a potential novel scaffold-target combination for development of new drugs against this remarkable pathogen and perhaps against other bacterial pathogens.

## MATERIALS AND METHODS

### Strains and growth medium.

All bacterial strains and growth conditions used in this study are outlined in Text S1 in the supplemental material.

### Enzyme expression and purification.

All recombinant proteins used in this study were derived from genes ligated into plasmid pET28a+ and expressed in and purified from Escherichia coli BL21(DE3) using standard techniques. In all cases, recombinant proteins were coexpressed with chaperone proteins (GroESL) to enhance solubility. His tags were cleaved with thrombin (except for *Mt*MurI, M. tuberculosis UDP-MurNGly-l-Ala:d-Glu ligase [*Mt*MurD], and *Mt*DapF) and protein preparations flash frozen and stored at −80°C. Thawed aliquots used for experiments were stored at 4°C and discarded after 2 to 3 days. All purified recombinant enzymes showed high (>95%) purity except *Mt*MurD (∼85% to 90%) and *Mt*DapF (75% to 80%). For more details, see Text S1 in the supplemental material.

### Enzyme assays.

Unless otherwise stated, all purified enzyme assays were performed at 37°C and monitored spectrophotometrically (340 nm) by coupling enzyme activity to reduction or oxidation of NAD(H) through a secondary enzyme(s) (see Text S1 in the supplemental material for more details). Exceptions were steady-state kinetic analysis of Bacillus subtilis MurI (*Bs*MurI) (circular dichroism) and activity testing of *Mt*DapF (liquid chromatography-mass spectrometry [LC-MS]). In all cases, kinetic and inhibition parameters and constants were derived using nonlinear regression analysis within SigmaPlot software (see Text S1).

Irreversibility of inactivation was tested by incubating 5 μM enzyme with various concentrations of BCDA (including zero-drug controls) in 50 mM HEPES (pH 7.6) for 4 h at 37°C. Samples were then dialyzed three times (40-fold dilution factor) into fresh 50 mM HEPES (pH 7.6) at 4°C and protein concentrations measured by bicinchoninic acid (BCA) assay. Enzyme activity was then measured as described for the steady-state kinetic analysis of Alr but employing 10 mM l-Ala and 5 nM recombinant enzyme. The percentage of remaining activity was then calculated by comparing the newly measured rate to that of the respective enzyme's zero-drug control.

### Microbiology.

MICs were measured by the broth microdilution method. Metabolomics data sets were generated by extracting polar metabolites from H37Rv or B. subtilis grown atop filters on solid media using an acetonitrile:methanol:water (ACN:MeOH:H_2_O) mixture and analyzing metabolite content through normal phase high-performance LC (HPLC) and accurate mass time of flight (ToF) MS, essentially as previously described (5, 12). For detection of cytoplasmic UDP-linked peptidoglycan intermediates by LC-MS, an acid hydrolysis step was added as previously described (13). See Text S1 in the supplemental material for more details.

### Proteomics.

Protein samples for proteomic analysis were prepared by incubating enzyme (10 to 60 μM) with (or without) BC(d/l)A (10 to 50 mM)–50 mM HEPES (pH 7.6) for 3 to 4 h at 37°C (6 h for *Mt*Alr) followed by extensive dialysis into 20 mM triethanolamine hydrochloride (TEA) (pH 7.8). Confirmation of loss or retention of enzyme activity was tested using standard coupled spectrophotometric assays as described in the supplemental material. Intact molecular weights (MWs) were determined by electrospray ionization-mass spectrometry (ESI-MS), and the site of adduct formation was determined by tryptic digest of protein samples followed by LC-tandem MS (LC-MS/MS). See Text S1 for more details.

Whole-cell H37Rv protein lysates for proteomic analysis were prepared by harvesting 100 ml of exponential-phase untreated or BCDA-challenged (2× MIC, 24 h) H37Rv (optical density [OD], 0.5 to 1.0) and resuspending the reaction mixture in 2 ml Tris- and phosphate-buffered 8 M urea (pH 7.4) containing 10% glycerol and 4 mM TCEP (Tris[2-carboxyethyl] phosphine hydrochloride). Cell suspensions were lysed by several rounds of ribolysis with intermittent cooling on ice. Triton X-100 and sodium deoxycholate were added to reach a 1% (wt/vol) final concentration, and the mixture was incubated on ice for 1 h with intermittent gentle mixing, followed by centrifugation at top speed for 10 min in a refrigerated microcentrifuge. The soluble fractions were passed through a 0.22-μm-pore-size spin filter, flash frozen, and stored at −80°C until further analysis. A 50-μg volume of this protein mixture was run in a single lane of an SDS-PAGE gel and the area corresponding to the MW of *Mt*MurI excised and trypsin digested prior to targeted MS detection of BCDA-modified *Mt*MurI as described in Text S1 in the supplemental material.

## RESULTS

### BCDA is a poor inhibitor of *Mt*Alr *in vitro*.

For PLP-dependent enzymes undergoing inactivation by BCDA, an important parameter that defines inhibitor potency is the partition ratio, defined as the number of 2-AA hydrolysis events (nonenzymatic conversion to pyruvate) relative to enzyme inactivation events (covalent, irreversible modification of enzyme). We overexpressed and purified recombinant forms of the B. subtilis and E. coli Alr enzymes and found that their partition ratios compared well with previously published values (Fig. 1C) (7–9, 14). In contrast, recombinant *Mt*Alr displayed a partition ratio substantially higher than those for E. coli Alr (*Ec*Alr) and *Bs*Alr (85- and 240-fold, respectively). This difference was also evident when partition ratios were compared for the structurally and mechanistically similar Alr inhibitor O-acetyl–d-serine (OADS). A more comprehensive analysis of the time dependence of inhibition of the three Alr enzymes by BCDA demonstrated that *Mt*Alr underwent inactivation at rates 940- and 1,700-fold lower (*k*_inact_ values) than those seen with the *Bs*Alr and *Ec*Alr orthologues, respectively, despite almost identical affinity values (*K_i_*, 130 to 230 μM) for BCDA (Fig. 1B and C; see also Fig. S1B in the supplemental material). Steady-state kinetic parameters of each Alr orthologue with respect to l-alanine are included in Fig. 1C for comparison (see also Fig. S1C). To confirm the irreversibility of inhibition by BCDA, protein samples treated with various concentrations of BCDA were extensively dialyzed into buffer lacking BCDA and subsequently tested for enzymatic activity. These results, shown in Fig. S1D, demonstrate the dose-dependent and irreversible effects of BCDA against all three enzymes, with the previously determined partition ratios correlating well with the respective concentrations of BCDA at which complete inhibition occurred (*Ec*Alr < *Bs*Alr < *Mt*Alr). Overall, these results reveal the differences in both catalytic processing of BCDA and enzyme reactivity to the 2-AA intermediate of *Mt*Alr relative to the B. subtilis and E. coli Alr orthologues.

### The d-alanine pathway is not the primary target of antibiotic action in M. tuberculosis.

**FIG 2:**
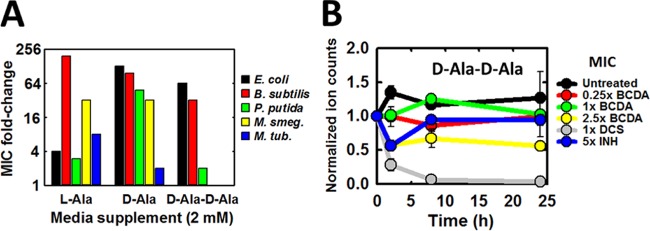
BCDA does not inhibit the d-alanine pathway in M. tuberculosis. (A) Fold increase in BCDA MIC relative to levels seen with unsupplemented control cultures in various bacterial species following growth medium supplementation with 2 mM l-Ala, d-Ala, or d-Ala-d-Ala. MICs were read 10 days following initial culture inoculation for H37Rv, 24 h following initial culture inoculation for E. coli, B. subtilis, and P. putida, and 48 h following initial culture inoculation for M. smegmatis (M. smegmatis). *M. tub*., M. tuberculosis. (B) Intracellular levels of d-Ala-d-Ala in H37Rv across 24 h of exposure to various test compounds (as indicated). Data for DCS and INH are taken from reference 5. Plots represent the averages ± SEM of data from duplicate experiments. See also Fig. S2 in the supplemental material.

The poor inhibitory potency of BCDA against *Mt*Alr *in vitro* suggested that this enzyme may not be the target of action of this compound in M. tuberculosis. In contrast to DCS, a known d-alanine pathway inhibitor, and in contrast to BCDA inhibition in other bacterial species (10), we found that supplementation of growth media with 2 mM d-Ala or d-Ala-d-Ala had either minor effects or no effects, respectively, on BCDA resistance in M. tuberculosis, while l-Ala had a stronger effect (Fig. 2A; see also Fig. S2A in the supplemental material). Next, we applied LC-MS-based metabolomics to H37Rv grown in the presence of BCDA to investigate the effects of drug treatment on intracellular levels of the dipeptide d-Ala-d-Ala, which, as previously shown by us and others, are depleted following DCS treatment and are generally indicative of d-alanine pathway inhibition. Intracellular BCDA levels increased proportionately with dose levels and were maintained in a constant manner throughout the studied time course (see Fig. S2B), suggesting that neither drug uptake nor metabolism is a limiting factor in the bacterial response to this compound. Strikingly, we saw no effect of BCDA treatment at 1-fold MIC on d-Ala-d-Ala levels over 24 h of drug exposure, while only a modest (∼50%) reduction was observed with 2.5-fold MIC treatment (Fig. 2B). This is in stark contrast to DCS treatment, where dipeptide levels are completely depleted after less than 12 h of drug exposure at 1× MIC (5).

Next, intracellular *Mt*Alr and *Mt*Ddl activities were monitored *in situ* following BCDA exposure using a metabolomics approach previously used by us in the delineation of DCS-target engagement in this bacterium (5). Using this method, we followed conversion of doubly labeled (α^2^H, 1× ^13^C) l-alanine (supplemented in growth media) to 1× ^13^C alanine (product of *de novo* Alr-catalyzed racemization) and 1× or 2× ^13^C d-Ala-d-Ala (product of *de novo* Ddl-catalyzed dipeptide formation) following an initial preexposure of M. tuberculosis to various concentrations of BCDA (see Fig. S2C in the supplemental material). While a clear inhibitory and dose-dependent effect of BCDA on *Mt*Alr and *Mt*Ddl activity was evident, low-level racemization and, more importantly, dipeptide formation persisted even at 2.5-fold MIC, in contrast to what was seen with DCS at equivalent doses (5). Together, these results imply that, while potentially partially responsible, the d-alanine pathway is not the primary target of antibiotic action of BCDA in M. tuberculosis.

### BCDA targets d-glutamate incorporation into peptidoglycan in M. tuberculosis.

**FIG 3:**
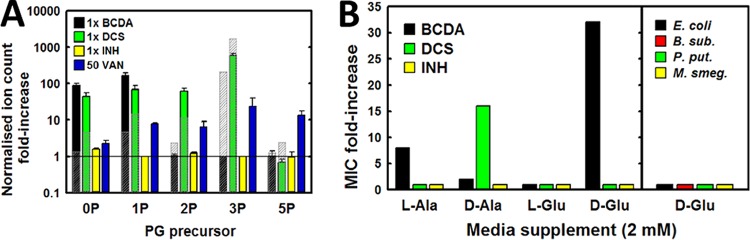
BCDA inhibits incorporation of d-Glu into nascent peptidoglycan precursors. (A) Fold change in intracellular levels of PG precursors in H37Rv, relative to untreated control cultures, following 24 h of challenge with various compounds at the specified concentrations. No MIC could be determined for vancomycin (VAN) using the filter-growth method, and therefore a concentration of 50 μg ml^−1^ was chosen based on published results (16). Translucent gray-shaded bars represent equivalent values measured in B. subtilis. Results are the averages ± SEM from at least duplicate independent experiments. (B) Fold change in MIC for H37Rv (left window) following supplementation of growth media with 2 mM l-Ala, d-Ala, l-Glu, or d-Glu. Data for l-Ala and d-Ala are taken from Fig. 2 and are shown here again for comparison. The right window shows the fold change in the BCDA MIC for four alternative bacterial species following supplementation of media with 2 mM d-Glu. See also Fig. S3 in the supplemental material.

We and others have previously demonstrated a strong synergy of antibiotic activity of DCS and BCDA against M. tuberculosis (5, 11). Synergy between compounds that target distinct enzymes within a single, essential metabolic pathway often occurs (15), and BCDA-DCS synergism has been identified by inhibition of Alr and Ddl, respectively (11). However, based on our observation of incomplete d-alanine pathway inhibition by BCDA as described above, we hypothesized that BCDA-DCS synergism may be due to BCDA inhibition of a separate enzyme within the cytosolic peptidoglycan biosynthetic pathway. Furthermore, the stereochemistry of BCDA suggests a target with matching stereo preference, and the multiple d-amino-acid-processing enzymes of peptidoglycan biosynthesis are strong candidates. To test this directly, we employed metabolomics to analyze the effects of drug treatment on the relative pool sizes of intracellular UDP-MurNAc-linked PG precursors. In agreement with previous publications (13, 16), the majority of soluble UDP-linked PG intermediates in H37Rv were found to be N-glycolylated (NGly) and not N-acetylated (NGly *m*/*z* value 16 mass units higher than those of N-acetylated derivatives; see Fig. S3A in the supplemental material). Therefore, all subsequent MS analyses of M. tuberculosis PG intermediates were based on the *m*/*z* values of MurNGly-linked peptides. Following BCDA treatment, we observed a depletion of all muropeptides from the dipeptide (L-Ala-d-Glu; 2P) onward and 100-fold to 300-fold increases in MurNGly and monopeptide (L-Ala; 1P) ion abundances compared to untreated control cultures (Fig. 3A). This is in contrast to DCS treatment, which induced an accumulation of tripeptide (L-Ala-d-Glu-*meso*-diaminopimelic acid [L-Ala-d-Glu-*meso*-DAP]; 3P), as expected for an inhibitor of d-Ala-d-Ala biosynthesis. This effect was not observed in B. subtilis, where BCDA treatment generated a PG precursor pool size profile very similar to that produced by DCS treatment. These data suggested that BCDA specifically targets incorporation of d-Glu into the growing muropeptide chain in M. tuberculosis. To test this hypothesis, we examined the effect of d-Glu supplementation in growth media on the MIC of BCDA. Indeed, the presence of 2 mM d-Glu in the growth media led to a 32-fold increase in the MIC for BCDA, a result that was not observed for DCS or for isoniazid (INH) (Fig. 3B). As a comparison, equimolar supplementation with l-Ala or d-Ala led to only 8- and 2-fold increases, respectively, in the MIC for BCDA. No effect was observed with 2 mM l-Glu supplementation, confirming that the protection provided by d-Glu did not arise from some inherent reactivity of the compound to glutamate. Also, d-Glu supplementation had no effect on the MIC of BCDA in any of the four alternative bacterial species tested (Fig. 3B).

We next tested the effects of d-Glu supplementation on intracellular PG precursor pool sizes following growth inhibition performed with BCDA. We used 1× ^13^C-labeled d-Glu in order to discriminate between *de novo* d-Glu incorporation and other forms of PG turnover or recycling. Over the 4-h time course of d-Glu supplementation (after an initial 24 h of BCDA exposure), the levels of 1× ^13^C–d-Glu-containing di- and tripeptide increased gradually from zero to ∼5,000 and 10,000 normalized ion counts, respectively. Equivalent normalized ion counts of unlabeled muropeptides in untreated bacteria were ∼1,000 and ∼200, respectively. Also, as shown in Fig. S3B in the supplemental material, little to no incorporation of labeled d-Glu into muropeptides occurred in bacteria not previously treated with BCDA. The increase in pool sizes for labeled d-Glu-containing pentapeptide precursors was not as evident as that for the di- and tripeptides; however, levels of this intermediate were consistently very low (and often below detection levels) under all conditions tested and at all time points. Also, d-Glu supplementation did not affect levels of intracellular BCDA (see Fig. S3C), indicating that d-Glu-mediated rescue was not due to competition with respect to substrate/inhibitor uptake. These results are consistent with BCDA inhibiting synthesis of d-glutamate and/or its incorporation into the UDP-linked substrate.

### BCDA is an irreversible-mechanism-based inhibitor of glutamate racemase.

**FIG 4:**
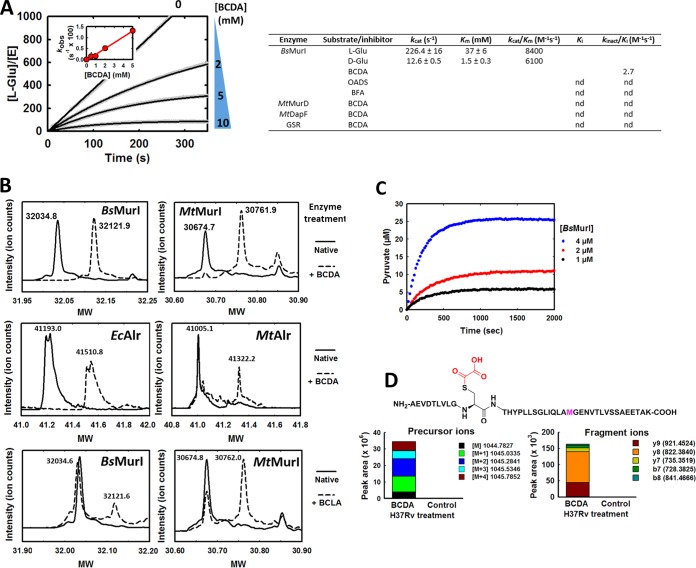
BCDA is an irreversible-mechanism-based inhibitor of glutamate racemase. (A) Time-dependent inhibition kinetics of BCDA against recombinant *Bs*MurI. Time courses displayed are representative of results of at least triplicate independent assays. The inset shows a replotting of *k*_obs_ versus BCDA concentration. Values in the table represent derived kinetic parameters for enzymes mentioned in this study (nd, not detected). GSR, S. cerevisiae glutathione reductase; BFA, β-fluoro-alanine (racemic mix); OADS, O-acetyl–d-serine. Data shown are the averages ± SEM of at least triplicate data sets. (B) ESI-MS analysis of intact molecular weights (MW) of untreated (solid lines) and BC(d/l)A-treated (dashed lines; 10 mM for 1 to 2 h, 37°C) recombinant *Bs*MurI and *Mt*MurI (top and bottom panels) and *Ec*Alr and *Mt*Alr (middle panels). (C) BCDA inhibition of *Bs*MurI proceeds via a 2-AA intermediate. Pyruvate production by *Bs*MurI in the presence of BCDA was measured using a coupled enzyme (pyruvate kinase/lactic dehydrogenase [PK/LDH]) assay. The BCDA concentration was held constant at 5 mM. Data displayed are representative of time courses selected from replicate data sets. (D) Local amino acid sequence corresponding to the predominant site of BCDA covalent modification in *Mt*MurI (top) and identification of the BCDA-modified *Mt*MurI peptide in whole-cell lysates derived from BCDA-treated (2× MIC, 24 h) H37Rv cultures, but not untreated control cultures, by LC-MS (bottom). The precursor ions (left panel) refer to the quadruple charge state (+4) of the parent peptide; the right panel displays the fragment ions derived from this precursor ion. Fragments were continuously monitored and were absent from the control sample. See also Fig. S5 and S6 in the supplemental material.

We next sought to identify the specific enzyme target of BCDA, with the most likely candidates being glutamate racemase (MurI) and UDP-MurNGly-l-Ala:d-Glu ligase (MurD), the two enzymes that directly employ d-Glu as a substrate. Unfortunately, we were unable to obtain measurably active forms of either recombinant *Mt*MurI or M. smegmatis MurI (*Ms*MurI), despite multiple attempts and diverse purification/expression strategies (see Text S1 in the supplemental material for details). This is in agreement with a recent report showing that multiple site-specific amino acid substitutions along the homodimer interface of *Mt*MurI are required to obtain measurable catalytic activity (17). We therefore performed all *in vitro* reactions with the B. subtilis orthologue (*Bs*MurI), an enzyme that shares 40% amino acid sequence identity (ID) and 56% sequence similarity (SIM) with *Mt*MurI. The steady-state kinetic parameters of our recombinant *Bs*MurI were in agreement with previously published data (18; values are listed in Fig. 4A). Importantly, BCDA displayed dose- and time-dependent inhibition of *Bs*MurI (Fig. 4A), suggestive of irreversible inhibition. Indeed, BCDA-treated *Bs*MurI did not regain catalytic activity following extensive dialysis. Replotting the observed *k* (*k*_obs_) values obtained from each time course against the BCDA concentration provided a *k*_inact_/*K_i_* value of 2.7 M^−1^ s^−1^. Two analogues of BCDA, β-fluoro-alanine (BFA; racemic mixture) and OADS, had no effect on *Bs*MurI activity at a final inhibitor concentration of up to 10 mM or following extended incubation of enzyme and inhibitor prior to activity testing (results not shown). In contrast, BCDA had no significant inhibitory activity against *Mt*MurD, including when enzyme was incubated for prolonged periods in the presence of BCDA and other reaction components (see Fig. S4 in the supplemental material). These results implicate MurI and not MurD as the target for BCDA in M. tuberculosis.

MurI is a PLP-independent racemase and is therefore unable to undergo the same mechanism of inhibition as Alr with BCDA. We therefore took a protein mass spectrometry approach to investigate the precise molecular mechanism of MurI inactivation by BCDA. The intact molecular weights of untreated and BCDA-treated *Bs*MurI were measured using ESI-MS and were found to differ by 87 (Fig. 4B, top left panel). This is in contrast to Alr, where BCDA treatment leads to an increase in molecular weight of 317 (19) (Fig. 4B, middle panels), corresponding to the canonical covalently modified and irreversibly bound 2AA-PLP-enzyme complex. Further analysis of BCDA-treated *Bs*MurI identified a single cysteine residue (C185; see Fig. S5 in the supplemental material) that was the sole site of modification in >95% of the inactivated protein. MurI is a member of the “two-base” mechanism (dual-cysteine) racemase family, where two essential active-site cysteine residues act as catalytic base and acid to stereospecifically de- and reprotonate, respectively, the alpha position of glutamate in order to enact substrate racemization (20). C185 of *Bs*MurI corresponds to the essential catalytic cysteine residue that deprotonates an incoming l-glutamate substrate (or reprotonates a carbanionic intermediate to form the d-stereoconfiguration). To probe the stereospecificity of this reaction, we performed ESI-MS analysis of *Bs*MurI treated with the enantiomer of BCDA, BCLA (β-chloro-l-alanine). As expected, BCLA modified *Bs*MurI predominantly at C74, the cysteine residue responsible for deprotonation of an incoming d-Glu substrate (Fig. 4B, bottom left panel; see also Fig. S5). Further tests revealed that BCDA had no inhibitory activity against *Mt*DapF, an epimerase that interconverts l,l-DAP and *meso*-DAP by employing a two-base mechanism and is also involved in PG biosynthesis (21), or against glutathione reductase, a model cysteine-thiol-dependent enzyme (22) (results not shown), suggesting that BCDA inhibition of MurI is mechanism based as opposed to arising from nonspecific interaction with suitably configured cysteine thiols.

Next, we investigated whether the molecular mechanism of inactivation proceeds via a 2-AA intermediate (as seen for Alr inactivation by BCDA) by testing for the presence of pyruvate in *Bs*MurI-BCDA mixtures. Indeed, pyruvate formation was detected using a coupled assay system following incubation of *Bs*MurI with BCDA (Fig. 4C), the rate of which followed first-order kinetics and was directly proportional to the concentration of enzyme employed (calculated partition ratio of 5.89 ± 0.59).

### BCDA modifies *Mt*MurI *in vitro* and *in vivo*.

Finally, we sought proof, independently of recombinant enzyme activity, that *Mt*MurI was similarly inactivated by BCDA, particularly within the whole-cell environment. Serendipitously, our seemingly catalytically inactive recombinant preparation of *Mt*MurI formed the +87 covalent adduct upon exposure to both BCDA and BCLA, as observed by ESI-MS intact molecular weight analysis (Fig. 4B, top and bottom right panels). The site of modification by BCDA was mapped to the cysteine residue at position 185 (see Fig. S6A in the supplemental material), identically to that seen with *Bs*MurI. Crucially, this BCDA-modified peptide was also identified in whole-cell lysates from bacteria previously treated with growth-inhibitory concentrations of BCDA but not in those from untreated control cultures (Fig. 4D; see also Fig. S6B to E). Furthermore, induced overexpression of *Bs*MurI in H37Rv led to a 16-fold increase in the MIC for BCDA compared to an empty vector control (see Fig. S6F), without affecting sensitivity to DCS or INH. *Mt*Alr overexpression also raised the MIC of BCDA by 8-fold. Despite confirmation of transgene expression by immunoblotting, *Mt*MurI overexpression had no discernible effect on the MIC of BCDA in H37Rv. Due to the inability to detect *Mt*MurI catalytic activity from either E. coli-derived recombinant enzyme or H37Rv cell lysates, we did not pursue this experimental setup any further. In any case, the protection afforded by *Bs*MurI overexpression with respect to the growth-inhibitory properties of BCDA provided further evidence that MurI is the primary target of antibiotic action of this compound in M. tuberculosis.

## DISCUSSION

### Essentiality and drugability of *Mt*MurI.

We initially began working with BCDA under the assumption that it was an Alr-specific inhibitor in M. tuberculosis and could therefore aid in differentiating the roles of Alr and Ddl in the mechanism of action of DCS, a clinically approved antituberculosis drug and major subject of research in our laboratory. However, as we have demonstrated in this paper, BCDA is in fact a very poor inhibitor of *Mt*Alr both *in vitro* and in whole cells and instead enacts M. tuberculosis growth arrest primarily through inhibition of glutamate racemase (MurI), a distinct enzyme within the peptidoglycan precursor biosynthetic pathway. MurI, a PLP-independent amino acid racemase, has been extensively studied as a potential broad-spectrum antibiotic target due to its conserved essentiality across almost all bacterial species, as well as its critical role in peptidoglycan biosynthesis, a well-validated and successfully exploited antibiotic target (for a review of glutamate racemase drug development, see reference 23). However, clinically effective MurI-specific antibiotics have yet to be realized. Several chemical scaffolds have been demonstrated to inhibit species-specific glutamate racemases *in vitro*, and enzyme inhibitory activity has been correlated and associated with antibacterial activity in a few of these cases (24–26). However, to the best of our knowledge, this is the first report of chemical inhibition of MurI in M. tuberculosis and associated bacterial growth inhibition. This both validates *Mt*MurI as a chemically accessible antibiotic target and permits further chemical genetic investigations of MurI function within M. tuberculosis.

### *Mt*Alr versus BCDA.

BCDA has been well studied as a mechanism-based inhibitor of a variety of PLP-dependent enzymes, and in all cases the mechanisms of inhibition have been presumed to be identical (Fig. 1A). Partition ratios have been measured in many cases and have ranged from 160 to 1,500 (7–9, 14, 27). While our measured values for *Ec*Alr and *Bs*Alr fit this range, the measured value for *Mt*Alr (>25,000) is a clear outlier. Esaki and Walsh have previously shown that partition ratios among diverse 2-AA-releasing β-substituted alanines are similar for an individual enzyme, and they conclude that this value is therefore an indicator of the reactivity of the shared intermediate 2-AA, rather than of the parent compound, to a specific active-site architecture (8). Our results determined with OADS support this hypothesis (Fig. 1C). This suggests the existence of substantial conformational or electrostatic differences between the active sites of *Mt*Alr and other orthologues that are not apparent in the available crystal structures (28). This is further substantiated by the differences between the steady-state kinetic parameters of the Alr orthologues, with the *Mt*Alr *k*_cat_/*K_m_* being more than 20- and 50-fold lower than the corresponding values for *Bs*Alr and *Ec*Alr, respectively. *k*_cat_/*K_m_* values correlate positively with *k*_inact_/*K_i_* values, while partition ratios correlate negatively with *k*_inact_ and *k*_inact_/*K_i_* or *k*_cat_/*K_m_* values, suggesting a link between catalytic processing (α-proton abstraction) and reactivity of the active site to 2-AA. However, Esaki and Walsh found the opposite in their characterization of catabolic (DadB) and anabolic (Alr) alanine racemases from Salmonella enterica serovar Typhimurium, suggesting that this correlation may simply be a coincidence that corresponds to our choice of experimental enzymes rather than a general rule (8). Expanding the repertoire of Alr enzymes used in this study should provide greater understanding of the properties that dictate partitioning of 2-AA between enzyme inactivation and hydrolysis. To investigate this phenomenon further, we have begun comparative structural and activity-based investigations of *Mt*Alr and other Alr orthologues, relating in particular to interactions with the clinically relevant antibiotic and Alr inhibitor DCS. Our preliminary results support the idea of the existence of substantial differences between the kinetic and mechanistic properties of *Mt*Alr that may be exploited for future targeted drug design studies.

While inhibition of *Mt*Alr may not be significantly relevant in the mechanism of action of BCDA, the high partition ratio observed for *Mt*Alr and BCDA predicts the release of high levels of 2-AA into the bacterial cytosol during drug treatment and raises the issue of whether this reactive intermediate has damaging effects outside Alr and MurI in M. tuberculosis. In fact, our data do not preclude an involvement of 2-AA generated by Alr in the inactivation of MurI in addition to the mechanism-based inhibition described here. 2-AA is known to react with and inactivate multiple PLP-dependent enzymes, both *in vitro* and when enzymatically overproduced within the cellular environment of various bacterial species, most notably essential transaminases involved in amino acid biosynthesis (19, 29, 30). Our observation that l-Ala supplementation, but not d-Ala supplementation, had a moderate rescuing effect on BCDA-mediated growth inhibition of M. tuberculosis could therefore be explained by 2-AA-mediated inactivation of alanine-requiring transaminases, such as transaminase C, which is involved in branched-chain amino acid biosynthesis (31). Assessment of the effects of supplementation of a broader range of amino acids on BCDA sensitivity could reveal more about additional indirect targets. As an aside, lack of rescue by d-Ala also suggests that competition with respect to uptake by the joint l/d-Ala transporter CycA, inhibition of Alr itself, or incorporation of BCDA into mature PG either via the cytoplasmic route or via periplasmic transpeptidases is insignificant in the mechanism of action of BCDA (32, 33). Recent research has identified a dedicated detoxification system—the RidA protein—involved in the removal of 2-AA intermediates produced during normal cellular metabolism (34) and has shown that, in *S*. Typhimurium, BCDA-derived 2-AA inhibition of the branched-chain amino acid aminotransferase IlvE is significant only in the absence of RidA enzymatic activity (30). M. tuberculosis carries two orthologues of the *S*. Typhimurium RidA protein, Rv2704 (28% ID, 52% SIM) and Rv3678c (26% ID, 43% SIM), neither of which has been biochemically characterized for 2-AA deaminase activity. Interestingly, only Rv3678c contains the essential Arg105 (*S*. Typhimurium RidA numbering) catalytic residue, suggesting an alternative role for Rv2704 (34–36). Furthermore, neither Rv2704 nor Rv3678c clusters with genes associated with natural 2-AA production or sensitivity, in contrast to what has been shown for many other RidA homologues (36). Further studies will demonstrate whether either of these RidA homologues functions in reactive imine deamination in M. tuberculosis, whether other detoxification systems exist, and whether M. tuberculosis is inherently sensitive to 2-AA production. The exploration of the use of reactive enamine/imine-generating prodrugs as an antituberculosis therapy warrants further investigation.

### *Mt*MurI versus BCDA.

**FIG 5:**
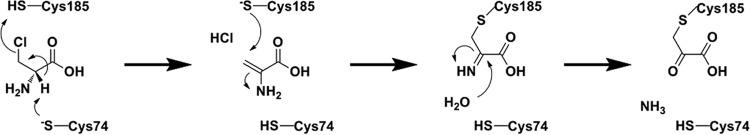
Plausible mechanism of covalent modification of MurI by BCDA. The cysteine numbering corresponds to the *Bs*MurI protein sequence.

Our results demonstrate that BCDA is a mechanism-based irreversible inhibitor of MurI and suggest that the mechanism of inactivation proceeds via a 2-AA intermediate (Fig. 5). Importantly, although active against recombinant *Bs*MurI, the antibiotic mechanism of action of BCDA in B. subtilis operates via Alr inhibition, as has previously been established for multiple bacterial species (10). The difference between M. tuberculosis and other bacteria in the mechanism of action is therefore likely due to the differential sensitivities of the two enzymes (Alr and MurI) to BCDA. For B. subtilis Alr and MurI, *k*_inact_/*K_i_* values of 790 and 2.7 M^−1^ s^−1^ (respectively) support this hypothesis; while we were unable to determine kinetic inhibition parameters for *Mt*MurI, it is probable that inhibition is more potent than it is for *Mt*Alr (1.5 M^−1^ s^−1^). Furthermore, Sengupta and colleagues have previously demonstrated extremely low catalytic activity for a urea-refolded recombinant form of *Mt*MurI, at levels at least 10-fold lower than that of *Ms*MurI, which itself already demonstrated low activity relative to other orthologues (37, 38). In fact, MurI enzymes in general display poor catalytic activity relative to other amino acid racemases (*k*_cat_/*K_m_* values of 1,000 to 10,000 M^−1^ s^−1^ [39]). MurI (in particular, *Mt*MurI) could therefore be a highly sensitive target due to poor affinity to and low catalytic activity for native substrates, making even moderate inhibitors of the enzyme (such as BCDA) highly active at the whole-cell level. Studies have also shown that intracellular d-Glu levels are perpetually low in various bacterial species, indicating low MurI activity and possibly highlighting a metabolic pool sensitive to perturbation by enzyme inhibition (40–44). In contrast to the results of Sengupta et al., Poen and colleagues have recently shown that *Mt*MurI adopts a unique oligomeric configuration and contains distinct active-site architectural differences from other MurI orthologues and that dimer interface mutations are required to introduce catalytic capability (17). Conventional MurI inhibitors are inactive against this recombinant form of mycobacterial MurI, with all the relevant results together suggesting that the selective inhibitory activity of BCDA against *Mt*MurI may arise from unique protein-ligand interactions unseen in orthologous MurIs. However, *Ms*MurI results demonstrate these same structural features and yet BCDA does not appear to act via MurI in this bacterium (see Fig. 2A). Additional parameters, including inhibitor uptake and the relative contribution of *Ms*Alr inhibition, may play a role in this case. Further studies will be required to clarify these issues.

A potential issue for BCDA, particularly if administered in combination with DCS, is acquirement of spontaneous resistance; we have shown here that overexpression of Alr is sufficient to significantly raise the MIC of BCDA in M. tuberculosis. As this is also a mechanism for gaining resistance to DCS in other bacteria (41, 45, 46), it is possible that a single mutation could diminish the effectiveness of the two drugs simultaneously. Therefore, any possible drug development based on the BCDA/2-AA-releasing scaffold would need to, first, reduce affinity and catalytic processing by Alr and, second, increase the affinity for MurI. The most obvious route would be substitution of the β-chloride with alternative leaving groups. We have already shown that β-fluoro and β-O-acetyl groups are not tolerated; however, a previous study demonstrated that l-serine-O-sulfate is processed by and, ultimately, inhibits Pediococcus pentosaceus MurI in a mechanism most likely akin to that described here (47). Although no follow-up mechanistic or microbiological studies were performed with this compound, the structural similarity of a β-sulfate group to the carboxylic acid side chain of glutamate may indicate higher affinity for MurI than for the much smaller chloride substituent. Changing the stereochemistry to the d-isomer would also likely increase the affinity and selectivity of this compound for MurI.

In summary, the data presented here demonstrate a novel mechanism of antibiotic action of the established Alr inhibitor BCDA in M. tuberculosis, where it arrests growth through covalent inhibition of the glutamate racemase MurI. These results therefore establish MurI as a bona fide and accessible drug target within this persistent pathogen, as well as highlighting significant catalytic differences between M. tuberculosis alanine racemase and other Alr orthologues that may be exploitable for drug design.

## Supplementary Material

Supplemental material
