# Supplementary material for: Glutamate Racemase Is the Primary Target of β-Chloro-d-Alanine in Mycobacterium tuberculosis
Source: Antimicrob Agents Chemother. 2016 Sep 23;60(10):6091–9. doi: 10.1128/AAC.01249-16 (PMC5038272; doi:10.1128/AAC.01249-16)
Supplement: Supplemental material [file supp_60_10_6091__index.html]

Glutamate Racemase Is the Primary Target of β-Chloro-d-Alanine in Mycobacterium tuberculosis — Supplemental material 

# Glutamate Racemase Is the Primary Target of β-Chloro-d-Alanine in Mycobacterium tuberculosis

## Supplemental material

- Supplemental file 1 -

  Supplemental experimental protocols and Figures S1 to S6

  PDF, 1.1M
