# Supplementary material for: Glutamate Racemase Is the Primary Target of β-Chloro-d-Alanine in Mycobacterium tuberculosis
Source: Antimicrob Agents Chemother. 2016 Sep 23;60(10):6091–9. doi: 10.1128/AAC.01249-16 (PMC5038272; doi:10.1128/AAC.01249-16)
Supplement: Supplemental material [file AAC.01249-16_zac010165571so1.pdf]

Supplemental Data

Glutamate racemase is the primary target of β-chloro-D-alanine in *Mycobacterium tuberculosis*

Gareth A. Prosser[1#*], Anne Rodenburg[1], Hania Khoury[1], Cesira de Chiara[1], Steve Howell[2], Ambrosius P. Snijders[2] & Luiz Pedro S. de Carvalho[1]*

[1] Mycobacterial Metabolism and Antibiotic Research Laboratory, and [2] Proteomics Scientific Technology Platform, Mill Hill Laboratory, The Francis Crick Institute, London, UK.

SUPPLEMENTAL EXPERIMENTAL PROTOCOLS

Strains and growth conditions

M. tuberculosis H37Rv was routinely grown in, and all liquid broth experiments performed in Middlebrook 7H9 medium supplemented with 0.2 % glucose, 0.2 % glycerol, 0.085 % NaCl, 0.05 % tyloxapol and 0.05 % BSA (c7H9) as described previously (1). For growth and experiments performed on solid media, c7H10 was used (Middlebrook 7H10 medium supplemented identically as described above). For transgene over-expression in H37Rv, the gene of interest was PCR amplified from gDNA, cloned into the tetracycline-inducible expression plasmid pTetR3 as a PacI-EcoRI fragment and electroporated into H37Rv along with the integrase-encoding plasmid pBSInt, as previously described (2). Positive transformants were selected on and maintained in media containing 25 µg ml^{-1} kanamycin. When required, gene expression was induced with 0.5 µg ml^{-1} anhydrous tetracycline (for MICs tetracycline was added at the time of the initial bacterial inoculation).

B. subtilis (subsp. *subtilis 168*), *E. coli* (BL21) and *Pseudomonas putida* (KT2440) were routinely grown in LB medium and MIC assays performed in a modified M9 minimal media (cM9; 1 × M9 salts, 1 × trace metals, 0.5 % glucose, 0.1 mM $CaCl_2$, 2 mM $MgSO_4$, 4 mM L-glutamate and 0.2 mM L-tryptophan, pH 7.2). *M. smegmatis* (mc^2155) was grown in and MIC determinations performed in c7H9 medium.

Recombinant protein purification

All genes used in this study were PCR amplified from genomic DNA (*M. tuberculosis* H37Rv, *E. coli* BL21, *B. subtilis* subsp. *subtilis* 168, *M. smegmatis* mc^2155) and cloned into the IPTG-inducible expression plasmid pET28a+ with N-terminal His-tag extensions. The coding sequence used for *Mt*Alr was an N-terminal truncated version (commencing at Met25 relative to the annotated sequence) to reflect the more likely start codon (3). Mycobacterial

enzymes (*Mt*Alr, *Mt*MurI, *Ms*MurI *Mt*MurD and *Mt*DapF) were co-expressed with the mycobacterial GroEL homologue Cpn60.2 and *E. coli* GroES chaperone proteins using the plasmid pTrc60.2-p15a (plasmid pTrc-60.2-GroES (4) modified in our lab to carry the p15a origin of replication). *Bs*DAAT (D-amino acid transaminase), *Bs*Alr, *Ec*Alr and *Bs*MurI were co-expressed with the *E. coli* GroEL-ES chaperone proteins using the plasmid pGro7 (Takara Bio). Sequence-verified constructs were transformed into *E. coli* BL21 (DE3) and bacterial cultures were selected for and maintained in LB medium containing relevant antibiotics. Unless otherwise stated, recombinant protein expression was achieved in ZYM5052 auto-induction medium (5) containing 100 μg ml^{-1} kanamycin and either 200 μg ml^{-1} ampicillin (pTrc60.2-p15a) or 34 μg ml^{-1} chloramphenicol (pGro7). L-arabinose was also added to strains harbouring the pGro7 plasmid (0.5 mg ml^{-1}) and pyridoxine (1 mM) was added to Alr-expressing cultures. Cultures, typically 1 l, were incubated at 37 °C, 240 rpm until mid-exponential phase (OD_{600} ~1) and thereafter at 18 °C, 240 rpm (overnight, ~20 h). Cells were pelleted by centrifugation (4000 rpm, 30 min, 4 °C) and resuspended in 50-100 ml lysis buffer (20 mM TEA, 500 mM NaCl, 50 mM imidazole, 10 mM $MgCl_2$, 10 % glycerol, 1 mM DTT, 1 mg ml^{-1} lysozyme, 0.5 % CHAPS, 2 U ml^{-1} TURBO DNAse (Life tech), 1 × Complete protease inhibitor (Roche), pH 7.8) per litre of culture. For Alrs, 1 mM pyridoxal phosphate (PLP) was also added. Cells were lysed by sonication (with intermittent cooling on ice) and soluble and insoluble fractions separated by centrifugation at 20 000 g for 1 h, 4 °C. Soluble fractions were passed through a 0.45 μm filter and applied to a 5 ml HP HisTrap nickel sepharose column (GE Healthcare) pre-equilibrated with Buffer A (20 mM TEA, 500 mM NaCl, 10 % glycerol, 50 mM imidazole, pH 7.8). The column was washed with 15 CV of buffer A and fractions collected following gradient elution with buffer B (20 mM TEA, 500 mM NaCl, 10 % glycerol, 500 mM imidazole). 1 mM DTT was also added to all running, dialysis and storage buffers for purification of *Bs*MurI, *Mt*MurI and *Mt*DapF to

protect critical active site cysteine residues. All chromatographic steps were performed on an AKTA prime FPLC system (GE Healthcare) at 4 °C. Peak fractions (as determined by absorbance at 280 nm) were collected, analysed by SDS-PAGE for purity and selected fractions dialysed three times (at least 100 times buffer volume) into 20 mM TEA, 150 mM NaCl, pH 7.8. PLP was added to 1 mM final concentration to all PLP-dependent enzymes prior to dialysis. Thrombin (Restriction grade, GE Healthcare) was added to a final concentration of 5 U ml^{-1} and samples were rocked gently at 4 °C for up to 24 h (degree of cleavage monitored by SDS-PAGE). Thrombin was removed by passing the sample through a pre-equilibrated 5 ml heparin sepharose column (GE Healthcare) (20 mM TEA, 150 mM NaCl; 0.1 M glycine was also added for PLP-containing enzymes due to strong interactions between PLP and heparin sepharose resin). The flow through was collected and analysed for remaining thrombin activity using fluorogenic thrombin substrate III (CalBioChem) Due to low yields, *Mt*MurI, *Ms*MurI, *Mt*MurD and *Mt*DapF were left uncleaved. Samples were then dialysed three more times to remove the cleaved His-tag (20 mM TEA or 20 mM $NaPO_4$, 150 mM NaCl, pH 7.8), concentrated, flash frozen and stored at -80 °C. *Mt*MurD was subjected to an additional size exclusion chromatographic step prior to this stage. Expression and purification of *Mt*MurI was also attempted in *E. coli* BL21 with a C-terminal His-tag, an N-terminal thioredoxin tag, or as the native unmodified enzyme, in *M. smegmatis* $mc^2$4517 with an N-terminal His-tag (plasmid pYUB28b; (6)), or from *E. coli* inclusion bodies (as previously described (7)) without success (no measurably catalytically-active enzyme produced).

Enzyme activity measurements and analysis

For steady-state kinetic analysis of Alrs, enzyme activity was monitored by UV-Vis spectrophotometry in the L-Ala to D-Ala direction using *Bs*DAAT and lactate dehydrogenase (LDH) as a coupling enzyme. Reactions contained 50 mM HEPES pH 7.6, 5 mM α-

ketogluratate, 0.25 mM NADH, 18-28 U ml^{-1} LDH (used as a mixture of pyruvate kinase/lactate dehydrogenase for convenience; Sigma), 4 µM BsDAAT, and varying concentrations of L-Ala. Reactions were started upon addition of enzyme (1-10 nM) and absorbance monitored at 340 nm (ε_{340nm} = 6,220 cm^{-1} M^{-1}).

For steady-state kinetic analysis of *Bs*MurI, enzyme activity was monitored by circular dichroism (CD) at 215 or 225 nm on a Jasco J-815 spectropolarimeter (ε_{215nm} = 13 deg mol^{-1}, ε_{225nm} = 3.4 deg mol^{-1}). Reactions consisted of 10 mM $NaPO_4$ buffer pH 7.5, 1 mM DTT, varying concentrations of L- or D-Glu and 80 nM BsMurI.

Calculation of steady-state kinetic parameters (K_m, k_{cat}) was achieved by fitting initial rate data with the Michaelis-Menten equation.

Kinetics of Alr inactivation by BCDA were measured by stopped flow (*Ec*Alr and *Bs*Alr) or standard (*Mt*Alr) UV-vis spectrophotometry by detecting pyruvate formation from BCDA, with reactions consisting of 50 mM HEPES pH 7.6, 18-28 U ml^{-1} LDH, 0.25 mM NADH, 100 nM (*Bs*Alr and *Ec*Alr) or 3 nM (*Mt*Alr) enzyme, and varying concentrations of BCDA (0.1-4 mM). Reactions were monitored at 340 nm for 4 hours (MtAlr) or until no more decrease in absorbance could be detected (*Ec*Alr and *Bs*Alr).

Kinetics of inactivation of *Bs*MurI by BCDA and other compounds were monitored at 340 nm in reactions consisting of 50 mM HEPES pH 7.6, 5 mM NAD+, 1 mM D-Glu, 5-10 U ml^{-1} L-glutamate dehydrogenase (LGDH; Sigma), 80 nM *Bs*MurI and varying concentrations of test inhibitor (0.5-10 mM). The partition ratio for BCDA with *Bs*MurI was measured by detecting pyruvate formation from BCDA in reactions consisting of 50 mM HEPES pH 7.6, 18-28 U ml^{-1} LDH, 0.25 mM NADH, 1-4 µM *Bs*MurI and 1-5 mM BCDA. Reactions were

monitored until no more decrease in absorbance was observed. The partition ratio was calculated as described for Alrs (see below).

For analysis of enzyme inactivation by BCDA (Alrs, *Bs*MurI), individual time courses were fit with a single exponential function (equation 1, where 'y' is concentration of product, 'a' is product concentration at time ∞, 'b' is the k_{obs} and 'x' is time) to derive values for k_{obs} at each concentration of BCDA tested.

$$y = a(1 - e^{-bx}) \qquad \text{Equation 1}$$

For *Mt*Alr time courses, the background rate of NADH oxidation was significant relative to the enzyme catalysed rate and was therefore subtracted from each measured time course (background rates were measured for each BCDA concentration tested in the absence of enzyme, and were linear in all cases). K_i and k_{inact} values were then calculated from either a linear (*Bs*MurI; k_{inact}/K_i parameter only; equation 2) or hyperbolic (Alrs; equation 3) fit of k_{obs} versus BCDA concentration plots, where 'y' is k_{obs}, 'a' is k_{inact}, 'b' is K_i, and 'x' is BCDA concentration.

$$y = a/b.x \qquad \text{Equation 2}$$

$$y = (a.x)/(b + x) \qquad \text{Equation 3}$$

Partition ratios (molar ratio of pyruvate produced per enzyme inactivation event) for each Alr with BCDA were calculated from the same inactivation time courses by dividing the parameter 'a' derived from equation 1 by the enzyme concentration employed. Partition ratios were confirmed by running additional reactions with varying enzyme concentrations and by analysing aliquots of the above reactions by LC-MS and comparing pyruvate levels to standards.

Activity measurements for *Mt*MurD consisted of 50 mM HEPES pH 7.6, 10 mM $MgCl_2$, 20 mM KCl, 1.5 mM PEP, 0.25 mM NADH, 9-14 U ml^{-1} LDH, 6-10 U ml^{-1} pyruvate kinase (PK), 0.1 mM UDP-MurNAc-1P, 2 mM ATP and 2 mM D-Glu, and varying concentrations of BCDA (1-10 mM). Reactions were started upon addition of enzyme (1 µM) and monitored at 340 nm. To interrogate time-dependent inhibition, reactions were commenced by addition of D-Glu, following 30 minutes pre-incubation of the remaining reaction mixture.

*Mt*DapF activity reactions consisted of 25 mM Tris-Cl pD 8.0, 5 mM racemic diaminopimelate, 0.25 or 2.5 mM BCDA, and 1 µM enzyme in 100 % D_2O. Aliquots were taken at several time points up to 20 minutes following commencement of reaction, diluted 20-fold into acidic ACN and analysed by LCMS.

Glutathione reductase (GSR) activity reactions consisted of 50 mM HEPES pH 7.6, 0.25 mM NADPH, 0.3 mM oxidised glutathione (GSSG), 0.01 U ml^{-1} GSR (Sigma) and varying concentrations of BCDA. Reactions were monitored spectrophotometrically at 340 nm.

MIC measurements

H37Rv was inoculated to a final OD_{600} of 0.01 in 100 µl of c7H9 in 96-well microplates containing serial dilutions of compound and any necessary supplements. Plates were incubated at 37 °C for 10 days prior to visual inspection for colony growth. The MIC was defined as the lowest concentration of test compound that completely inhibited bacterial growth. MIC experimental layout for other bacterial species was identical to that described above for *M. tuberculosis*, except for growth media requirements (see above) and that MICs were visually inspected after 16 h growth at 37 °C for *E. coli* and *B. subtilis* and after 48 h growth for *P. putida* and *M. smegmatis*.

Metabolomics

Metabolomic analysis of H37Rv and *B. subtilis* was carried out essentially as previously described (1, 8). Briefly, 1 ml of exponential phase H37Rv or *B. subtilis* (OD_{600} ~ 1.0) was inoculated onto a single 25 mm 0.22 um pore size nitrocellulose filter under vacuum pressure, placed bacteria-side up onto c7H10 or cM9 solid medium (4 per 90 mm dish) and incubated at 37 °C for 5 days (H37Rv) or 4 hours (*B. subtilis*). Bacteria-laden filters were then transferred to fresh c7H10 or cM9 plates containing necessary investigational supplements and incubated at 37 °C. At various time points (90 minutes only for *B. subtilis*) biomass was removed from filters using a cell scraper and deposited directly into 1 ml of -40 °C acetonitrile:methanol:water (ACN:MeOH:H2O; 2:2:1 v/v) mixture, kept at temperature on dry ice. Biomass and solvent were transferred to 2 ml screw cap polypropylene vials containing ~300-400 µl of acid-washed 150 µm glass beads, ribolysed for 2 × 30 seconds at setting 6.5 (5 minutes cooling on dry ice in between times) and finally centrifuged at top speed, 4 °C, for 10 minutes. Supernatant was then spin-filtered through a 0.22 µm cellulose acetate filter and stored at -20 °C until ready for processing.

Prior to LCMS analysis, sample aliquots were mixed 1:1 with acidified ACN (0.2 % acetic acid). An Agilent 1200 LC system equipped with a solvent degasser, binary pump, temperature-controlled auto-sampler and temperature-controlled column compartment containing a Cogent Diamond Hydride Type C silica column (150 mm × 2.1 mm; dead volume 315 µl) was used for liquid chromatography. The flow rate on Agilent 1200 LC was 0.4 ml min^{-1}. The mobile phase method consisted of 0 min 85 % B; 0-2 min 85 % B; 2-3 min to 80 % B; 3-5 min 80 % B; 5-6 min to 75 % B; 6-7 min 75 % B; 7-8 min to 70 % B; 8-9 min 70 % B; 9-10 min to 50 % B; 10-11 min 50 % B; 11-11.1 min to 20 % B; 11.1-14 min hold 20 % B, where solvent A consists of deionized water, 0.2 % acetic acid and solvent B

consists in acetonitrile and 0.2 % acetic acid. An Agilent Accurate Mass 6230 TOF apparatus was used. Dynamic mass axis calibration was achieved by continuous infusion of a reference mass solution using an isocratic pump connected to a multimode ionization source, operated in the positive-ion and negative-ion mode. ESI capillary and fragmentor voltages were set at 3500 V and 100 V, respectively. The nebulizer pressure was set at 40 psi and the nitrogen drying gas flow rate was set at 10 L min^{-1}. The drying gas temperature was maintained at 250 °C. The MS acquisition rate was 1.5 spectra/sec and *m*/*z* data ranging from 50-1200 were stored. This instrument routinely enabled accurate mass spectral measurements with an error of less than 5 parts-per-million (ppm), mass resolution ranging from 10,000-25,000 over the *m/z* range of 121-955 atomic mass units, and a 100,000-fold dynamic range with picomolar sensitivity. Data were collected in the centroid mode in the 4 GHz (extended dynamic range) mode.

For measurement of cytoplasmic levels of UDP-linked PG intermediates ACN:MeOH:H_2O metabolite extracts were dried by speed-vac, resuspended in 50 µl 0.1 M HCl and boiled at 95 °C for 10 minutes. 10 µl was then transferred to 90 µl acidic ACN (0.2 % acetic acid) and centrifuged for 15 min at 13,000 rpm, 4 °C prior to analysis by LCMS (identical conditions as previously). Metabolite levels were normalized by comparison of alanine and lysine pool sizes within each sample before and after the extra processing steps described above. UDP-MurNAc-linked peptidoglycan standards were sourced from the BaCWAN facility at the University of Warwick, UK (9, 10). The MIC for BCDA used in metabolomics experiments was determined by observing cellular growth, after 5 days, of H37Rv inoculated onto filters and incubated on solid c7H10 media containing BCDA serial dilutions. The MIC under these conditions was determined to be 80 µg ml^{-1}.

Proteomics

Intact mass analysis

Protein molecular mass was determined using a micrOTOF-Q electrospray mass spectrometer (Bruker Daltonics, Coventry, UK). Protein was desalted using a 2 mm × 10 mm guard column (Upchurch Scientific, Oak Harbor WA) packed with Poros R2 resin (Perseptive Biosystems, Framingham). Protein was injected onto the column in 10 % acetonitrile, 0.10 % acetic acid, washed with the same solvent and eluted in 60 % acetonitrile, 0.1 % acetic acid. Desalted protein was then infused into the mass spectrometer at 3 µl min^{-1} using an electrospray voltage of 4.5 kV. Mass spectra were deconvoluted using maximum entropy software (Bruker Daltonics, Coventry, UK).

BCDA modification site analysis

Intact MW analysis revealed an 87 Da covalent adduct for *Bs*MurI and *Mt*MurI upon exposure to BCDA suggesting the modification of a single residue within these constructs. For the identification of the site of modification(s) by BCDA 1 µg of untreated or treated enzyme (taken from the same samples described above directly prior to dialysis) was run on SDS-PAGE. Peptides were generated using overnight in-gel trypsin digestion.

On an Ultimate 3000 nanoRSLC HPLC (Thermo Scientific) 1-10 µl of digested protein acidified to a final concentration of 0.1 % TFA was loaded at 5 µl min^{-1} of 0.1 % TFA onto a 2 mm × 100 µm Acclaim Pepmap C18 trap column (Thermo Scientific) prior to the trap being switched to elute at 0.3 µl min^{-1} through a 50 cm × 75 µm Acclaim Pepmap C18 column. A 70’ run with a gradient of 9 %-25 % B over 35', then 25 %-40 % B over 15' was used followed by a 4’ gradient to 100 % B and back down for equilibration in 9 % B (A= 0.1 % formic acid; B= 80 % ACN, 0.1 % formic acid).

Eluant was introduced into an LTQ Orbitrap Velos Pro (Thermo Scientific) via a Proxeon NanoES source (Thermo Scientific) fitted with a 30um ID stainless steel emitter operated at 2 kV. The Orbitrap was operated in "Data Dependent Acquisition" mode with a survey scan at a resolution of 60,000 from m/z 300-1500, followed by sequential CID and HCD MS/MS in the orbitrap of the top 3 ions. Dynamic exclusion was used with a time window of 20 s. The Orbitrap charge capacity was set to a maximum of 1e6 ions in 10ms, whilst the LTQ was set to 1e4 ions in 100 ms.

Raw files were processed using Proteome Discoverer (PD) 1.3 (Thermo Scientific) with Mascot 2.4 (Matrix Science, UK) as the search engine against the appropriate protein FASTA database. A decoy database of reversed sequences was used to filter the results, removing false positives, at a false detection rate of 1%. Label free quantitation was achieved using the precursor-ion quantitation module of PD.

Targeted method for the identification of BCDA modification in vivo

As the *Mt*MurI BCDA modification site determined by the above method was unlikely to be detected from the cell culture bands due to suspected low intensity, a targeted method was devised which would be compatible with analysis using Skyline software (MacCoss lab).

The site for MtMurI BCDA modification was found to be the cysteine within the tryptic peptide: AEVDTLVLGC*THYPLLSGLIQLAMGENVTLVSSAEETAK. DDA analysis showed the 4+ ion as the predominant precursor ion charge state and this information was used to set-up a targeted acquisition method on the LTQ-Orbitrap-Pro using Xcalibur software. The targeted method was set up to take the +/- BCDA modified versions of the peptide as well as methionine oxidation into account according to the below table.

Sequence	Mass/charge
AEVDTLVLGCTHYPLLSGLIQLAMGENVTLVSSAEETAK4+	1019.0260
AEVDTLVLGCTHYPLLSGLIQLAM(ox)GENVTLVSSAEETAK^{4+}	1023.0247
AEVDTLVLGC(BCDA)THYPLLSGLIQLAMGENVTLVSSAEETAK^{4+}	1040.7840
AEVDTLVLGC(BCDA)THYPLLSGLIQLAM(ox)GENVTLVSSAEETAK^{4+}	1044.7827

The Ultimate 3000 nanoRSLC HPLC was used as in the section above with a longer gradient as follows: A 120’ run with a gradient of 9 %-45 % B over 80', then 45 %-58 % B over 9' was used followed by a 10’ gradient to 100 % B and re-equilibration in 9 % B (A= 0.1 % formic acid; B = 80 % ACN, 0.1 % formic acid).

Eluate was introduced into the Orbitrap Velos Pro as above. The orbitrap was operated with an iterating hybrid scan selection consisting of parent ion scan followed by top 5 DDA CID and 4 narrow targeted HCD scans set to detect the target peptides (as detailed in the table above) at a collision energy of 35 and an MS/MS scan range of m/z 715 – 940 (to monitor y7, y8, y9 ions with b7 and b8 ions also occurring in this window

All ion extraction and peak area calculations were performed using Skyline software. Precursor ion masses were extracted from the MS1 scan whereas the y7, y8, y9, b7 and b8 ions were extracted from the targeted MS2 scans.

The method was tested initially using recombinant *Mt*MurI + BCDA, - BCDA, + BCLA and was shown to provide sufficient selectivity and sensitivity for the detection of *Mt*MurI BCDA modification (data not shown). The method was then applied to *M. tuberculosis* extracted proteins. Protein extracts were run on a SDS-PAGE and areas corresponding to the predicted MW of MurI were subjected to in gel trypsin digestion as described above. The presence of

MurI was confirmed using the DDA method (Figure S6). The BCDA modified peptide was detected as described above (Figure 4).

Figure S1

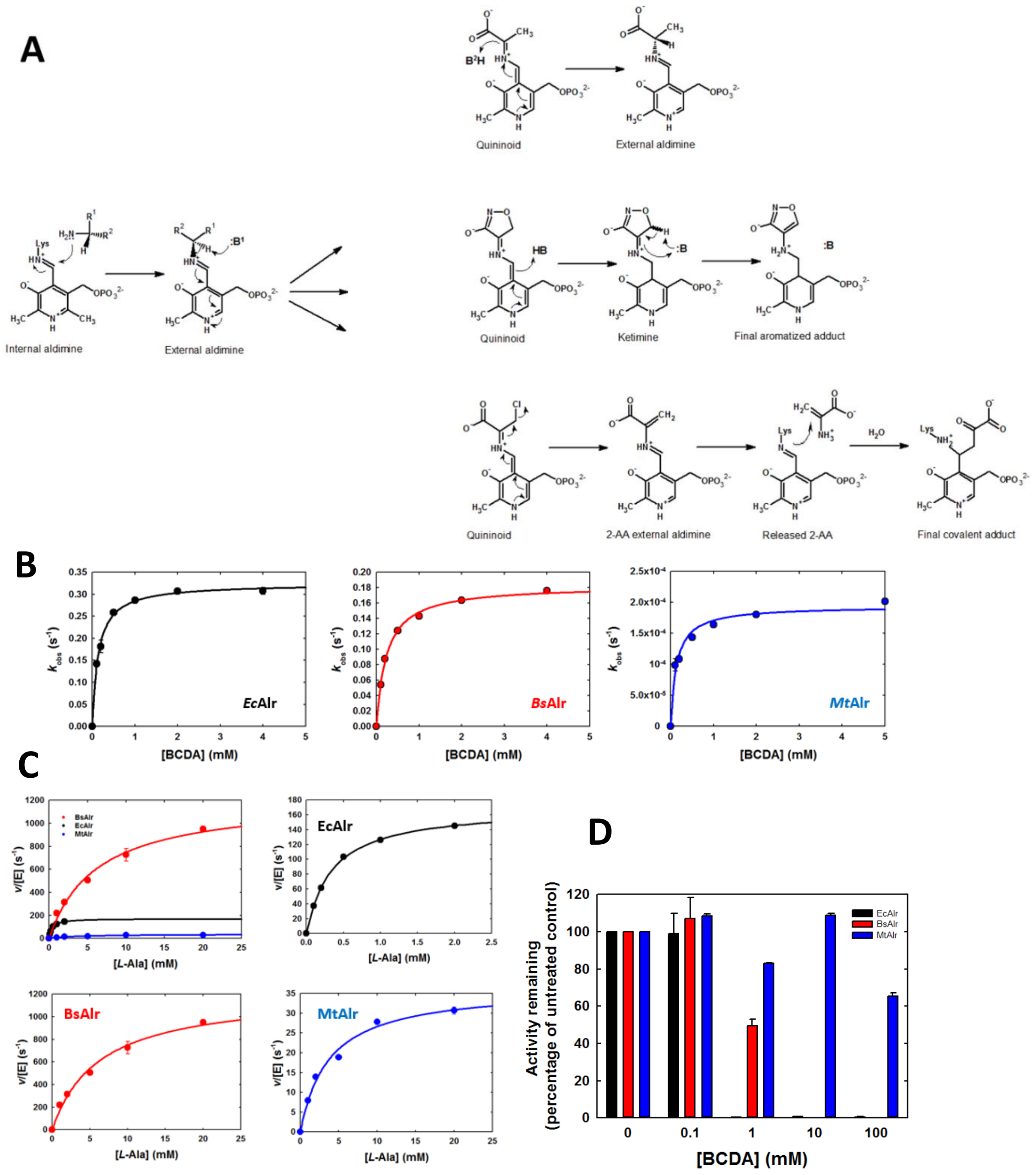

Figure S1. (A) Various known and predicted chemical mechanisms of PLP-substrate/inhibitor interactions. **(B)** Secondary plots of Alr inhibition by BCDA. k_{obs} values (as determined from fitting inhibition time-course data with equation 1) vs BCDA concentration for each Alr orthologue tested. Replots were fit with equation 3 to generate estimates for K_i and k_{inact} values. **(C)** Michaelis-Menten plots of Alanine racemase orthologues. Activity was measured in the L-Ala to D-Ala direction. All experiments were performed at pH 7.6, 37 °C, using a coupled enzyme assay system (*Bs*DAAT/PK). (**D**) BCDA irreversibly inactivates Ec- and BsAlr much more readily than MtAlr. Bars show Alr enzyme activity remaining, as a percentage of untreated controls, of enzyme samples (5 μM) exposed to BCDA (at the concentrations displayed) for 4 hours at 37 °C, followed by extensive dialysis to remove unbound BCDA. Activity was measured in the L-Ala to D-Ala direction using a *Bs*DAAT/PK coupled enzyme assay, as described in Experimental Protocols. All data is the average of at least 2 independent experiments ± SEM.

Figure S2

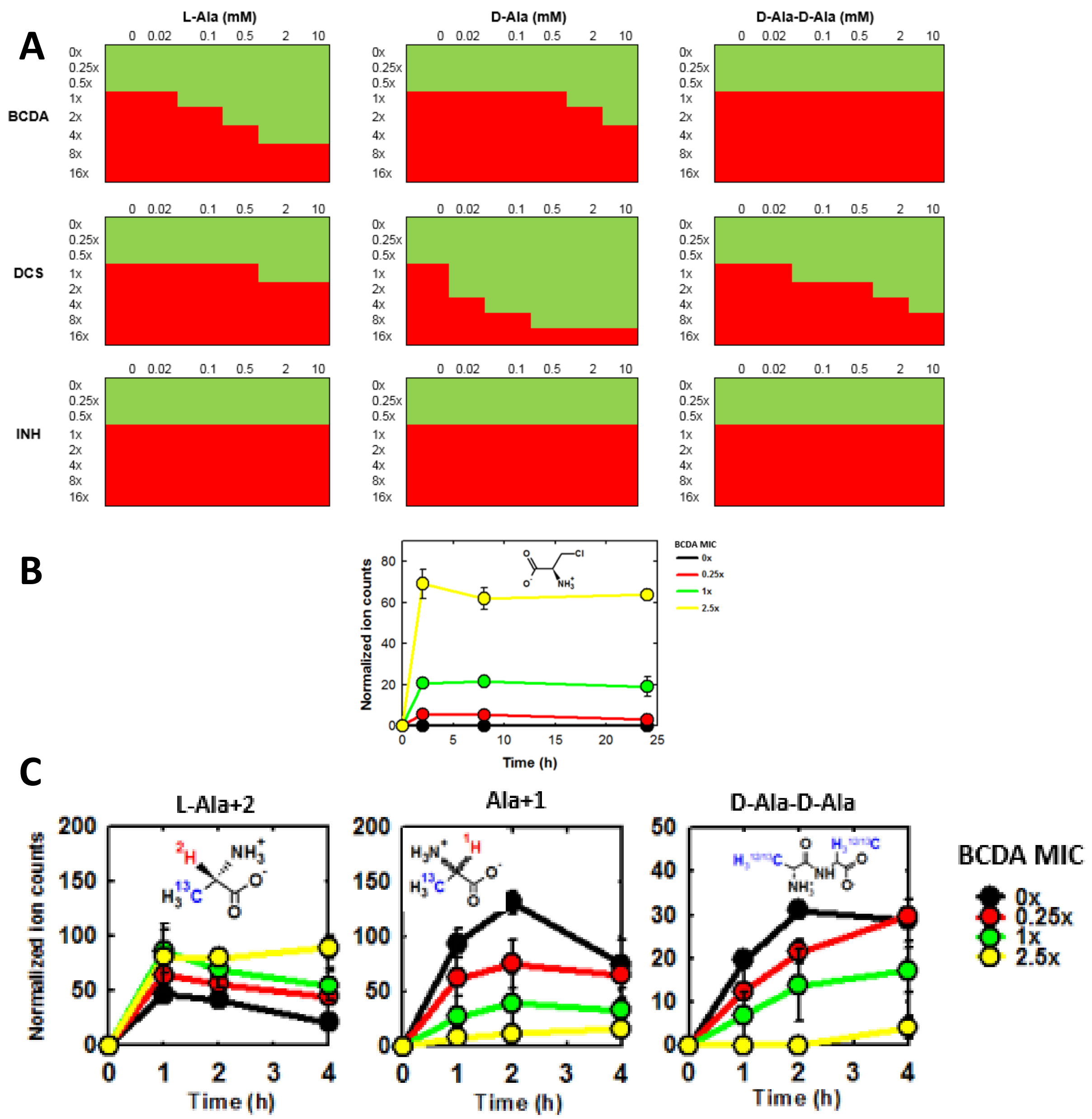

Figure S2. (A) D-Ala and D-Ala-D-Ala are poor phenotypic rescuers of BCDA sensitivity. Chequerboard assay demonstrating the effect of supplementing growth media with L-Ala, D-Ala and D-Ala-D-Ala on the MICs of BCDA, DCS, and INH in *M. tuberculosis* H37Rv. Values on the vertical axes indicate fold-increase in MIC over un-supplemented controls. Green shading signifies growth, red shading signifies no growth following a 10 day incubation. **(B)** BCDA levels were measured across 24 hours of drug exposure at multiple drug concentrations, using LCMS-based metabolomics as described in Materials and Methods. **(C)** Alanine racemase is poorly inhibited by BCDA in vivo. H37Rv intracellular levels of doubly-labelled L-Ala (α-^{2}H, 1× ^{13}C; left panel), singly-labelled D/L-Ala (α-^{1}H, 1× ^{13}C; middle panel) and all isotopologues of D-Ala-D-Ala (right panel) across a 4 hour period of 1× ^{13}C α-^{2}H L-Ala media supplementation (0.5 mM) following an initial 24 hours of BCDA exposure (at concentrations indicated). All values are the average ± SEM of at least 2 independent experiments.

Figure S3

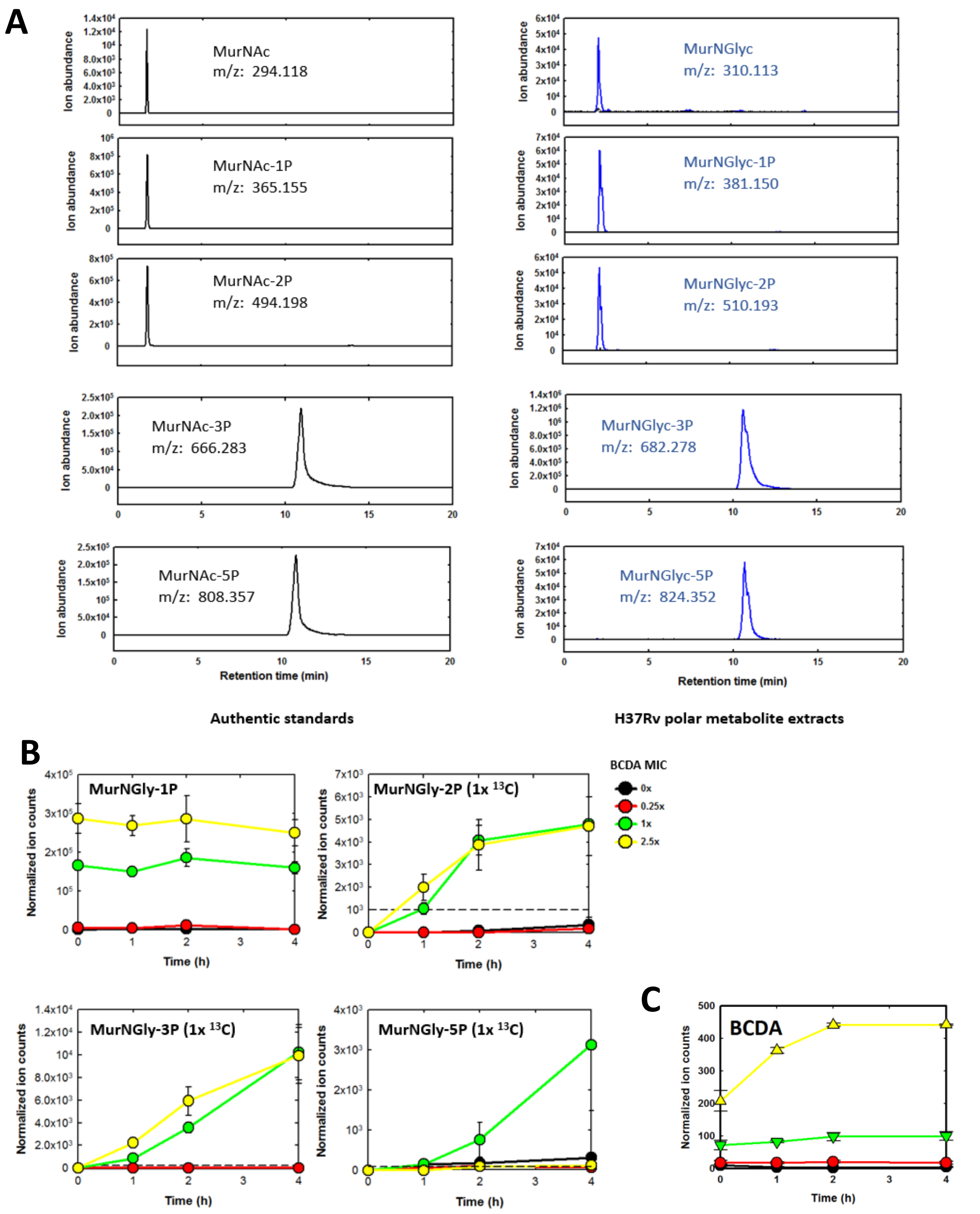

Figure S3. (A) Extracted ion chromatograms of MurNAc and MurNGlyc-linked PG intermediates. Extracted ion-chromatograms of MurNAc- (black) and MurNGlyc- (blue) linked PG precursors derived from either authentic MurNAc-linked standards (left column) or H37Rv cell extracts (right column), following hot acid-treatment and LCMS analysis as described in Experimental Protocols. **(B)** D-Glu supplementation rescues BCDA-induced PG intermediate depletion. Time course of incorporation of 1× ^{13}C D-Glu into PG precursors, as measured by LCMS, following supplementation of 24 hour BCDA-challenged filter cultures with a growth-rescuing concentration (2 mM) of the labelled substrate. Dashed lines indicate average levels of unlabelled peptides in untreated bacteria. All results are the average ± SEM of at least 2 independent assays. (C) Normalized ion counts of BCDA extracted from the same timecourses as shown in (B).

Figure S4

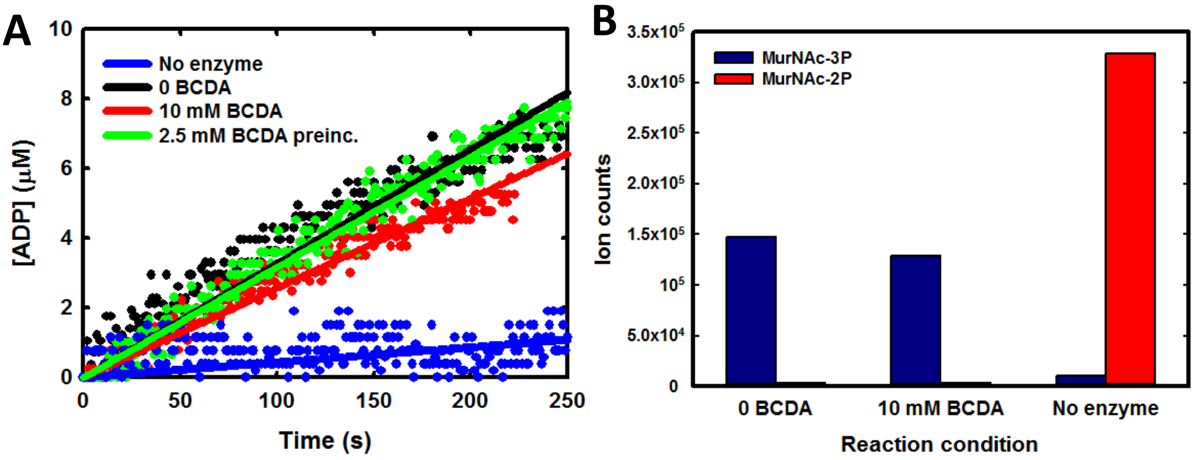

Figure S4: BCDA does not inhibit recombinant *Mt*- or *Bs*MurD. (A) Recombinant enzymes were tested for ATP-dependent UDP-MurNAc-L-Ala:D-Glu ligase activity in the presence and absence of BCDA using a spectrophotometric coupled enzyme assay (see Experimental Protocols). Timecourses are representative of at least triplicate assays. For 0 and 10 mM BCDA reactions timecourses were commenced upon addition of enzyme. For 2.5 mM preinc. reactions all components except D-Glu were preincubated for 30 minutes on ice prior to timecourse commencement upon addition of D-Glu. (B) Reactions described in (A) were allowed to progress for ¬4 h prior to hot acid treatment and LCMS analysis as described in Experimental Protocols. Extracted ion m/z values for the expected product (MurNAc-3P) and parental substrate (MurNAc-2P) are listed in Fig. S3. Data shown are from a single experiment.

Figure S5

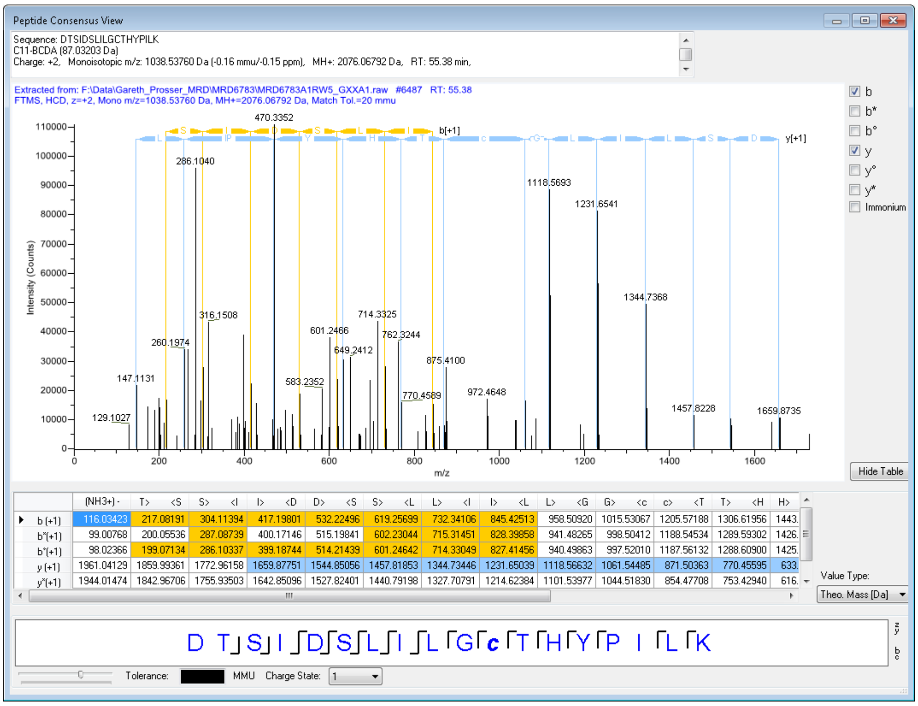

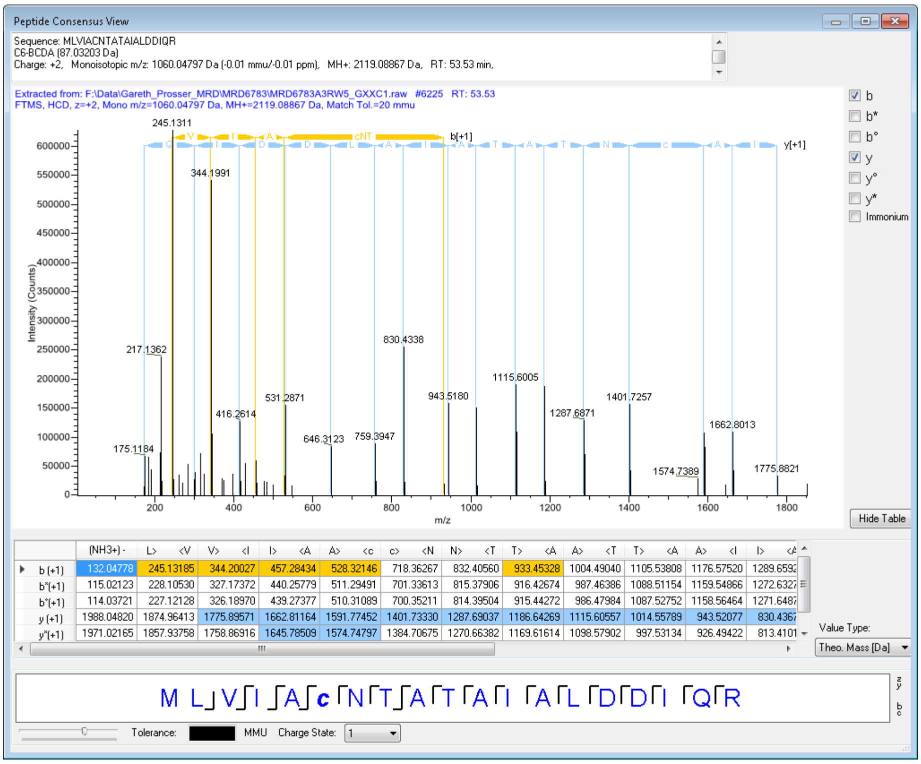

Figure S5. Annotated MS/MS spectra of tryptic peptides derived from BCDA- (top panel) and BCLA- (bottom panel) treated *Bs*MurI showing the precise site of covalent modification by BCDA. The modified cysteine (C185 for BCDA, C74 for BCLA) in each case is displayed as a lower case ***c***.

Figure S6

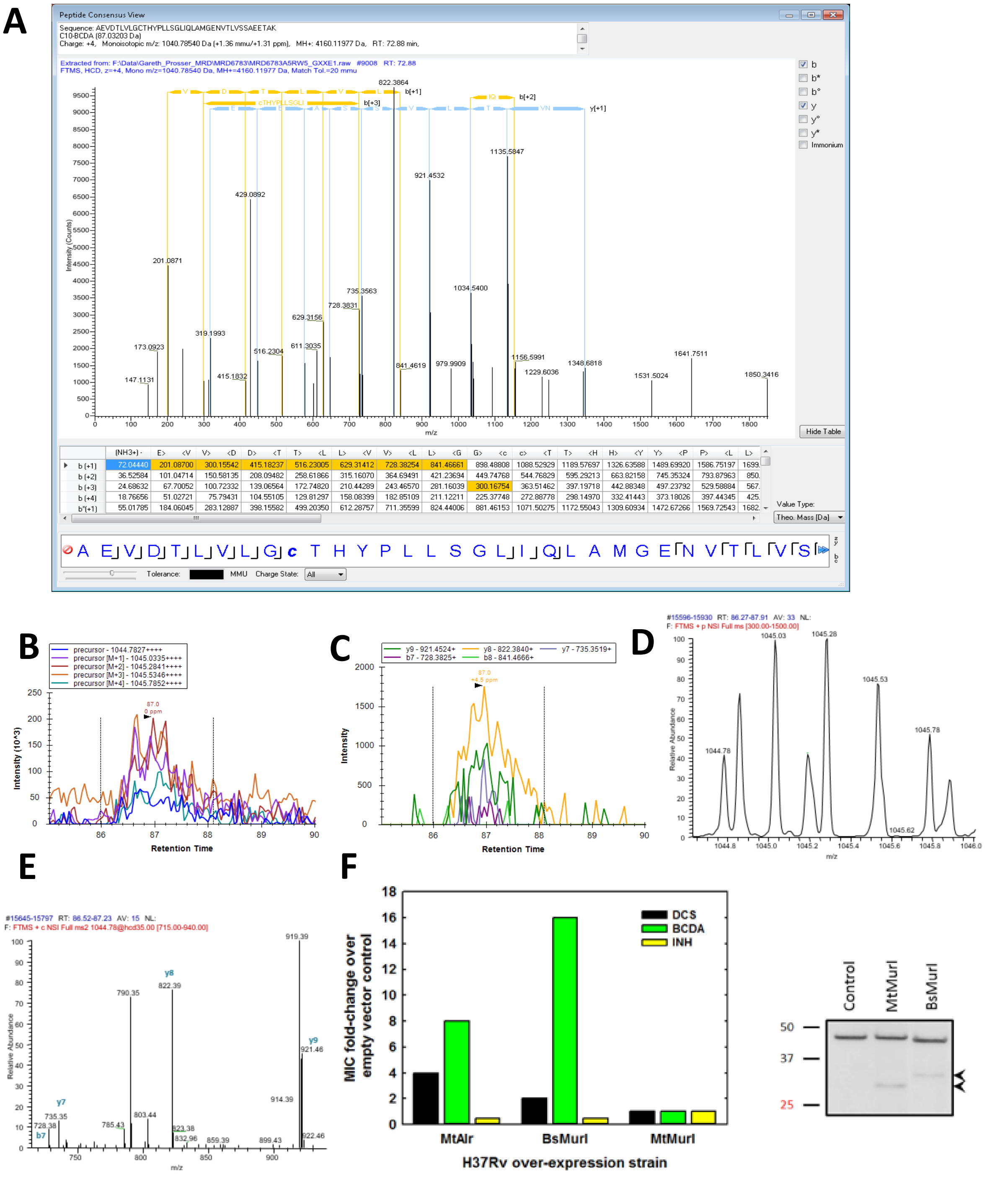

Figure S6. (A) Annotated MS/MS spectra of tryptic peptides derived from BCDA-treated *Mt*MurI showing the precise site of covalent modification by BCDA (lower case ***c*** in peptide sequence). **(B-E)** Identification of BCDA-modified MtMurI in whole cell lysates of BCDA-treated H37Rv. Extracted Ion Chromatograms (EIC) corresponding to the BCDA modified peptide AEVDTLVLGC(BCDA)THYPLLSGLIQLAM(ox)GENVTLVSSAEETAK^{4+} in BCDA treated H37Rv for (B) precursor ions and (C) fragment ions. Corresponding mass spectra of (D) precursor ions and (E) fragment ions are also displayed. (F) Over-expression of *Bs*MurI and *Mt*Alr, but not *Mt*MurI, increases the MIC of BCDA relative to an empty vector control strain. Left panel: Change in MICs of DCS, BCDA and INH, relative to empty-vector controls, following over-expression of either recombinant *Mt*Alr (native), *Bs*MurI or *Mt*MurI (both N-terminally His-tagged) in H37Rv. Results are the average of 2 independent assays. Right panel: Representative immunoblot showing location of hexa-histidine tagged recombinant proteins (arrows).

SUPPLEMENTAL REFERENCES

1. **Prosser GA**, **de Carvalho LPS**. 2013. Metabolomics Reveal d-Alanine:d-Alanine Ligase As the Target of d-Cycloserine in Mycobacterium tuberculosis. ACS Med Chem Lett **4**:1233–1237.

2. **Schuessler DL**, **Cortes T**, **Fivian-Hughes AS**, **Lougheed KEA**, **Harvey E**, **Buxton RS**, **Davis EO**, **Young DB**. 2013. Induced ectopic expression of HigB toxin in Mycobacterium tuberculosis results in growth inhibition, reduced abundance of a subset of mRNAs and cleavage of tmRNA. Mol Microbiol **90**:195–207.

3. **Strych U**, **Penland RL**, **Jimenez M**, **Krause KL**, **Benedik MJ**. 2001. Characterization of the alanine racemases from two mycobacteria. FEMS Microbiol Lett **196**:93–98.

4. **Batt SM**, **Jabeen T**, **Bhowruth V**, **Quill L**, **Lund PA**, **Eggeling L**, **Alderwick LJ**, **Fütterer K**, **Besra GS**. 2012. Structural basis of inhibition of Mycobacterium tuberculosis DprE1 by benzothiazinone inhibitors. Proc Natl Acad Sci U S A **109**:11354–11359.

5. **Studier FW**. 2005. Protein production by auto-induction in high density shaking cultures. Protein Expr Purif **41**:207–234.

6. **Bashiri G**, **Rehan AM**, **Greenwood DR**, **Dickson JMJ**, **Baker EN**. 2010. Metabolic Engineering of Cofactor F420 Production in Mycobacterium smegmatis. PLoS ONE **5**:e15803.

7. **Sengupta S**, **Shah M**, **Nagaraja V**. 2006. Glutamate racemase from Mycobacterium tuberculosis inhibits DNA gyrase by affecting its DNA-binding. Nucleic Acids Res **34**:5567–5576.

8. **Larrouy-Maumus G**, **Biswas T**, **Hunt DM**, **Kelly G**, **Tsodikov OV**, **de Carvalho LPS**. 2013. Discovery of a glycerol 3-phosphate phosphatase reveals glycerophospholipid polar head recycling in Mycobacterium tuberculosis. Proc Natl Acad Sci U S A **110**:11320–11325.

9. **Clarke TB**, **Kawai F**, **Park S-Y**, **Tame JRH**, **Dowson CG**, **Roper DI**. 2009. Mutational analysis of the substrate specificity of Escherichia coli penicillin binding protein 4. Biochemistry **48**:2675–2683.

10. **Lloyd AJ**, **Gilbey AM**, **Blewett AM**, **De Pascale G**, **Zoeiby A El**, **Levesque RC**, **Catherwood AC**, **Tomasz A**, **Bugg TDH**, **Roper DI**, **Dowson CG**. 2008. Characterization of tRNA-dependent peptide bond formation by MurM in the synthesis of Streptococcus pneumoniae peptidoglycan. J Biol Chem **283**:6402–6417.
